# Effect of Indoor Forest Bathing on Reducing Feelings of Fatigue Using Cerebral Activity as an Indicator

**DOI:** 10.3390/ijerph19116672

**Published:** 2022-05-30

**Authors:** Chie Imamura, Kiyomi Sakakibara, Kyosuke Arai, Hideki Ohira, Yuhei Yamaguchi, Hitoshi Yamada

**Affiliations:** 1 Toyota Central R&D Labs., Inc., Nagakute 480-1192, Japan; k-sakakibara@mosk.tytlabs.co.jp; 2 Frontier Research Center, Toyota Motor Corporation, Toyota 471-8572, Japan; kyosuke_arai@mail.toyota.co.jp (K.A.); yuhei_yamaguchi@mail.toyota.co.jp (Y.Y.); hitoshi_yamada_aa@mail.toyota.co.jp (H.Y.); 3 Department of Psychology, Nagoya University, Nagoya 464-8601, Japan; ohhira.hideki@a.mbox.nagoya-u.ac.jp

**Keywords:** indoor forest bathing, biophilic, brain activity, near-infrared spectroscopy, prefrontal cortex activity, reduction of feeling of fatigue

## Abstract

We created an indoor forest bathing environment in a sunlight-type environmentally controlled chamber and both physiological and psychological measurements were conducted for the evaluation of mental fatigue reduction. At first, a working memory load experiment was performed among 10 participants in a space without plants to identify an indicator correlating with feelings of fatigue, using the cerebral activity of the prefrontal cortex. Then, the indicator was used to evaluate whether a 20-min exposure to an indoor forest bathing environment reduced the level of the feeling of fatigue. The working memory load experiment demonstrated that, when mental fatigue increased, the amount of oxygenated hemoglobin (oxy-Hb) in the right prefrontal cortex and the right-left difference in oxy-Hb (ΔRL oxy-Hb) in the prefrontal cortex increased. These were proposed as indicators of mental fatigue. In the indoor forest bathing experiment, staying in an indoor green space showed that the subjective values of feeling of fatigue decreased and ΔRL oxy-Hb decreased. Since these results demonstrated an opposite effect to the increase in ΔRL oxy-Hb related to the feeling of fatigue, it was inferred that the decrease in ΔRL oxy-Hb reflected the fatigue reduction in the indoor forest bathing environment.

## 1. Introduction

Our ultimate goal is to develop technology for a space in which humans coexist with plants and microorganisms and results in well-being by incorporating elements of biophilic design [1]. Biophilic design aims to create artificial environments that resemble natural surroundings and bring health and wellbeing to people through the positive effects of nature [2,3]. We would like to apply the findings of research on indoor forest bathing to biophilic design.

Natural environments are known to relax and reduce stress. Studies on the effects of forest bathing and indoor plants on people have been reported [4,5,6,7,8]. Many indoor experiments [9] have been conducted, as preparing an experimental environment in nature and verifying the relationship between the psychological and physiological states are difficult. In indoor experiments, visual effects, such as placing a foliage plant in the office, viewing images of greenery and visual reality (VR), and odor effects attributed to forest components have been conducted as individual experiments. However, in indoor experiments, there are gaps in the types and number of plants and the sense of realism when compared with nature. Thus, comprehensively verifying their effect on human senses is challenging.

In this study, we constructed forest bathing environments in sunlight-type environmentally controlled chambers (biotron greenhouse, 3 × 3 × 5 m, width × height × depth) that reproduce plant groups similar to nature. This was considered for the following reasons: (1) There are several types of plants, and the green visibility from an individual’s point of view is more than 80%, based on the count from a photograph using a simple method. Moreover, the proximity to the plants is closer since they are planted within the reach of the individual’s seat. (2) As a biotron greenhouse can artificially control environmental conditions, such as temperature, humidity, and air change rate, it is possible to evaluate the effects of plants in the same environment. (3) Owing to the abundance of plants, it is possible to verify not only the visual sense but also the overall human senses, such as odor, and leaf fluctuation. In our experiment, the participants sat in the biotron greenhouse and were investigated for both physiological and psychological responses to verify the effect of an indoor forest bathing environment on mental fatigue reduction.

In a relaxed state, cerebral activity is hypothesized to calm an individual, resulting in a decrease in the cerebral blood flow. The concentration of oxygenated hemoglobin (oxy-Hb) in the prefrontal cortex changes when people view or interact with plants or other natural phenomena. In the evaluation of positive emotions, Song et al. reported the physiological effects of visual stimulation using forest imagery on the activity of the brain and autonomic nervous system [5]. The results showed that forest imagery induced increased feelings of comfort and relaxation, resulting in significant decreases in the oxy-Hb levels in the right prefrontal cortex. A similar effect was also reported when participants viewed fresh red roses [6]. Viewing roses increased feelings related to comfort and relaxation and significantly decreased the oxy-Hb levels in the right prefrontal cortex. In addition, touching hinoki cypress wood induced a similar increase in the feelings related to comfort and relaxation, along with significant decreases in the oxy-Hb levels in the left prefrontal cortex [7]. Furthermore, working for 3 min in an environment with foliage plants induces more physiological and psychological relaxation than working in an environment without foliage plants [8]. The result showed that the oxy-Hb concentration in the left prefrontal cortex decreased in the environment involving foliage plants. Therefore, although these experiments reported a decrease in the oxy-Hb concentration in the prefrontal cortex owing to observations of natural phenomena, such as plants or tactile stimuli, the effects on the left and right sides of the prefrontal cortex differed. Consequently, this study focused on the right-left difference in the oxy-Hb concentration (ΔRL oxy-Hb) in the prefrontal cortex.

Meanwhile, as an evaluation of negative emotions, right laterality ratio score (RLS = (Right − Left)/(Right + Left) of oxy-Hb and total hemoglobin) in the prefrontal cortex activity under stress has been reported [10,11]. We calculated RLS score using our data from the fatigue-inducing experiment, but there was no significant correlation between RLS score and the subjective values of feeling of fatigue. In this study, instead of the laterality ratio score, the right-left difference score (right-left) calculated from the absolute value of the oxy-Hb concentration in the prefrontal cortex was used, aiming for the direct evaluation of reducing feelings of fatigue.

Two types of fatigue have been identified: mental fatigue, caused by interpersonal relationships and concern, and physical fatigue. This study targeted mental fatigue, specifically short-term fatigue, which takes several minutes to several hours to recover from, or daily fatigue, which may require several days to recover from, rather than illnesses, such as chronic fatigue syndrome (CFS), which requires significantly longer recovery periods. The degree of fatigue is increased by fatigue factors. Conversely, the degree of fatigue can be reduced by the action of recovery factors [12]. Cases of short-term fatigue are generated by fatigue factors, such as temporary stress and fatigue-inducing tasks. However, natural phenomena, such as greenery, are considered candidates for recovery factors for this type of fatigue.

Tanaka et al. examined the autonomic nervous alterations associated with daily level of fatigue [12] by performing a fatigue-inducing two-back test [13] for 30 min, and immediately before and after the two-back test they evaluated fatigue using the 30-min advanced trail making test (ATMT) [14]. In this study, to reduce the long-term experimental workload of participants, we replaced the 30-min two-back tests with more challenging three-back tests for 20 min; furthermore, 30 min of ATMT was replaced with a 5-min psychomotor vigilance task (PVT) [15]. Using these fatigue-inducing tests, we examined the cerebral activity alterations in the prefrontal cortex to elucidate an indicator of the daily level of fatigue. Subsequently, we attempted to verify the hypothesis as to whether negative emotions, such as fatigue (unpleasant feelings, etc.), could be reversed by spending time in an indoor forest bathing environment, an environment similar to being in nature, using the ΔRL oxy-Hb in the prefrontal cortex.

## 2. Materials and Methods

### 2.1. Fatigue-Inducing Experiment

#### 2.1.1. Participants

Thirty-eight people aged between 20 and 40 years (18 men; average age ± standard deviation, 28.4 ± 8.1 years; 20 women; average age ± standard deviation, 29.4 ± 7.3 years) participated in this study. When filling out the informed consent form, participants confirmed that they had no major or pre-existing medical conditions such as cardiac disease, and no symptoms of hypertension, EEG abnormalities, autonomic system abnormalities, or asthma inflammation. Participants were also asked if they were feeling ill or had any subjective symptoms on the day of the experiment or within the past week. The cerebral blood flow of 10 of the 18 male participants (average age ± standard deviation, 27.3 ± 7.6 years) was measured using the TRS before and after the tasks. Two conference rooms with no plants—only tables and chairs—were used to conduct the experiment over 5 days, with four male and four female participants tested per day in separate rooms. The measurement was performed between 14:00 to 17:00 h after a break for lunch, and the duration was divided into two sessions of 1.5 h each. Participants were required to abstain from alcohol the day before the experiment. On the day of the experiment, the participants were instructed to eat the same meal for lunch between 11:30 and 12:00 h in the waiting room, rinse their mouths, and then take a break until 12:30 h. After lunch, only water was available until the end of the experiment. The aims and procedures of this study were fully explained to all participants.

#### 2.1.2. Study Protocol

**Figure 1:**
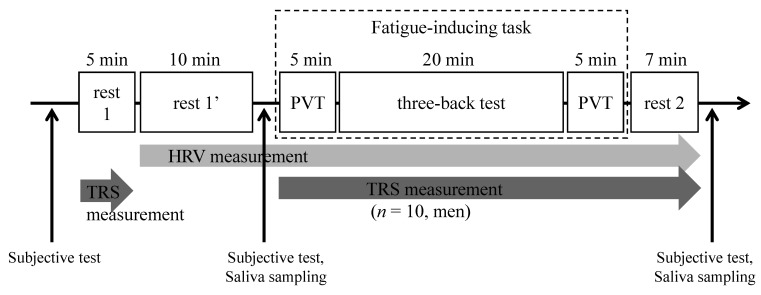
Study protocol. TRS, near-infrared time-resolved spectroscopy; HRV, heart rate variability; PVT, psychomotor vigilance task.

Figure 1 shows the sequence of the experiment. The fatigue-inducing experiment was performed in a conference room. First, baseline data for prefrontal cortex activity and heart rate variability (HRV) were collected for 5 min (rest 1) and 10 min (rest 1′), respectively, with the participant in a relaxed seated posture. Measurements were also performed with the participant in a relaxed seated posture after the fatigue-inducing task for 7 min (rest 2). Ten men from the 38 participants were assessed using TRS from the beginning of the first PVT to the end of the rest 2. HRV was measured using portable electrocardiography (BioSignalsplux; Creact, Tokyo, Japan). Subjective test and saliva sampling were performed between sessions. The experimental environments were measured using AM-101 amenity meters (Kyoto Electronics Manufacturing, Kyoto, Japan), and the temperature of the rooms was adjusted to maintain a predicted mean vote (PMV) value between −1 and 1. Although not discussed in this paper, saliva collection and HRV measurement were performed and are described in the study protocol.

#### 2.1.3. Fatigue-Inducing Task

A three-back test was conducted for 20 min to induce mental fatigue, with a PVT conducted before and after the three-back test to measure the behavioral indicator of fatigue. The three-back test involved memorizing letters from A to E displayed at random and pressing a button marked with a circle if the displayed letter was the same as the letter displayed third in the sequence or pressing a button marked with a cross if the letter was different. The participants were notified of an incorrect answer by a loud buzzer to generate stress and tension. For the PVT, the participants were instructed to press the screen rapidly once the figure zero displayed on the initial screen began to increase. All tasks were implemented on an iPad (Apple, Cupertino, CA, USA).

### 2.2. Indoor Forest Bathing Experiment

#### 2.2.1. Participants

The participants consisted of eight women aged between 30 and 45 years (average age ± standard deviation, 39.0 ± 5.4 years). When filling out the informed consent form, participants confirmed that they had no major or pre-existing medical conditions such as cardiac disease, and no symptoms of hypertension, EEG abnormalities, autonomic system abnormalities, or asthma inflammation. Participants were also asked if they were feeling ill or had any subjective symptoms on the day of the experiment or within the past week. Each test lasted for 4 days and was performed in one room each day. Measurements were performed between 13:00 and 15:30 h, with the test period divided into two 45-min sessions. Two participants were measured each day over a 16-day period. Counterbalancing was applied considering the entry sequences for the four rooms. Participants were required to abstain from alcohol the day before the experiment. On the day of the experiment, participants were instructed to eat the same meal for lunch from 11:00 to 11:30 h in the waiting room, rinse their mouths, and then take a break until 12:00 h. After lunch, only water was available until the end of the experiment. The aims and procedures of this study were fully explained to all participants.

#### 2.2.2. Study Protocol

**Figure 2:**
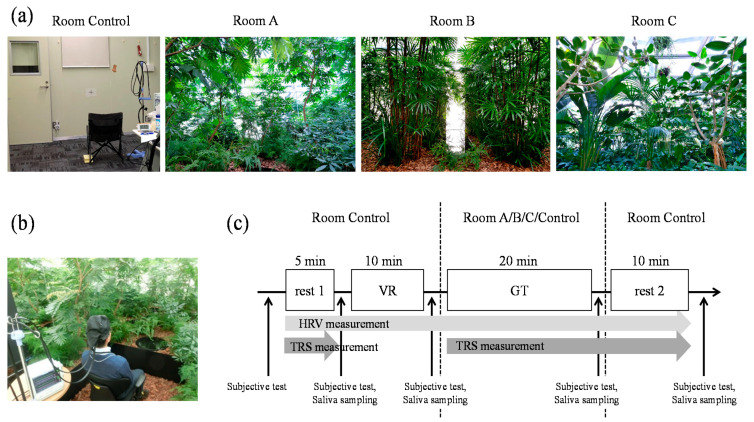
Experimental procedure. (**a**) Scene during staying spaces. (**b**) State at the time of measurement. (**c**) Study protocol. TRS, time-resolved spectroscopy; VR, visual reality; HRV, heart rate variability; GT, test space; Room Control, space without natural green features; Room A/B/C, space with natural green features.

To verify the effect of indoor rooms with different indoor greenery designs, we actually constructed three types of rooms in a biotron greenhouse with Pasona Panasonic Business Service Co., Ltd. The three rooms are called “Room A” “Room B” and “Room C”. “Room A” was created using a group of small leafy plants, “Room B” was created using a group of long and thin leafy plants, and “Room C” was created using a group of large and wide leafy plants. A space without natural green features (“Room Control”) was used as the negative control. Figure 2 shows the sequence of the experiment. To measure the baseline of the participants before entering the test space (GT), the participants were instructed to sit in a relaxed posture in Room Control for 5 min (rest 1). Subsequently, the participants were instructed to wear a VR system and play a shooting game (Rez Infinite, PlayStation 4; Sony, Tokyo, Japan) for 10 min as a light fatigue-inducing task (VR). Next, the participants entered the indoor green space and were instructed to sit for 20 min while quietly observing the plants in front of them. To enable measurement from the moment when the participants saw the green features, the participants were instructed to wear an eye mask before entering the space. The eye mask was removed, and the measurement was simultaneously started once the participant sat down. After this 20-min period, the participants returned to the Room Control, and the measurement was repeated for 10 min with the participants in a relaxed posture (rest 2). The processes for the negative control were performed entirely in Room Control. In Room Control, participants were instructed to watch the crosshair mark affixed to the height of the line of sight on the front wall from a distance of 1.5 m. In the case of the experiment in Room Control, there was no movement of the room, but the same tasks as when moving to the green space (standing up once, walking a few steps up the stairs to the space, going down four steps, wearing an eye mask next to the chair, and sitting down) were performed. Subjective test and saliva sampling were performed between the sessions. The experimental environments were measured using Midori Cloud (Seraku, Tokyo, Japan), and AM-101 amenity meters, and the temperature of the rooms was adjusted to maintain a PMV value between −1 and 1.

### 2.3. Psychological Measurement

Participants filled in the following four types of subjective tests as psychological indicators.

1. Feeling of fatigue: The visual analog scale (VAS) was used to evaluate the fatigue state of patients with CFS as a score from 0 to 100, from “not fatigued at all” to “extremely fatigued” [16]. In this study, to assess the fatigue of healthy participants instead of that of patients, the edge of the scale was modified to “no feeling of fatigue” and “feeling exhausted.”
2. Affect Grid [17]: This two-dimensional scale is used to evaluate how a participant is feeling. The participant selects an appropriate square in a 9 × 9 grid defined by arousing/sleepy on the vertical axis and pleasant/unpleasant on the horizontal axis.
3. Roken Arousal Scale (RAS) [18]: This is a subjective evaluation indicator of fatigue and arousal that consists of a six-factor emotional scale (sleepiness, activation, relaxation, strain (tension), difficulty maintaining attention and concentration, and lack of motivation). Each of these scales are evaluated using two questions, and an average score is calculated from seven levels (inapplicable to extremely applicable). The 12 items on the questionnaire include heavy eyelids and feeling sleepy for the sleepiness scale; being full of vitality and having a positive mood for the activation scale; feeling relaxed and restful for the relaxation scale; experiencing nervousness and throbbing for the strain (tension) scale; experiencing dullness and difficulty in concentration for the difficulty maintaining attention and concentration scale; and feeling unmotivated and reluctant to do anything for the lack of motivation scale. The scales of “activation” and “relaxation” were reordered to match the direction of the scale with the feeling of fatigue and were set as “unactive” and “unrelaxed.”
4. Phasic Stress Scale (PSS) [19]: The PSS is a subjective evaluation method for transient stress that consists of 18 questions. The questions are categorized into six emotional scales (malaise, pleasant, anger, anxiety, stress, strain). Each emotional scale is evaluated using one to four questions, and the average score is calculated. The 18 items in the questionnaire include looking lifeless, feeling depressed, and feeling empty for the fatigue scale; feeling refreshed, relieved, comfortable, and pleasant for the pleasantness scale; feeling angry, frustrated, exasperated, and disgruntled for the anger scale; feeling worried, unhappy, unstrung, and skittish for the anxiety scale; feeling stressed for the stress scale; and experiencing nervousness and throbbing for the strain scale. Each item is evaluated using an appropriate term along a VAS from totally inapplicable to extremely applicable. The scale of “pleasant” was reordered to match the direction of the scale with the feeling of fatigue and was set as “unpleasant.”

Other questionnaires relating to profile (age, occupation, number of working days), lifestyle (exercise, sleep), and causes of fatigue were also answered before the experiment.

### 2.4. Measurement of Cerebral Blood Flow Using Near-Infrared Time-Resolved Spectroscopy

Cerebral blood flow was measured using tNIRS-1 system (Hamamatsu Photonics, Shizuoka, Japan) as an indicator of brain activity. The sensors were mounted on the participant’s forehead and the concentration of oxy-Hb, deoxygenated hemoglobin (deoxy-Hb), total hemoglobin (tHb = oxy-Hb + deoxy-Hb), and tissue oxygen saturation (StO_2_ = oxy-Hb/tHb) in the left and right prefrontal cortex were measured. Because tNIRS-1 uses near-infrared time-resolved spectroscopy (TRS), it can produce quantitative data that can be compared between the participants and days. The sampling interval was 0.2 Hz.

### 2.5. Data Analysis

The measured TRS data were analyzed to calculate the following values: 0–1-min mean, mean value for the first minute immediately after the start of measurement; ΔRL, right-left difference (right-left) value of the 0–1-min mean; R/L ratio, ratio (right/left) of the 0–1-min mean; 0–5 min slope, slope over the first 5-min period. The amount of change before and after the task (rest 2–rest 1) was then calculated.

The following objective task result indicators were calculated: the accuracy in the initial and latter periods of the three-back test, the number of valid PVT responses before and after the three-back test (i.e., the number of responses in a 5-min period), and the PVT mean reaction time (from the start of the count to touching the screen).

Paired *t*-tests, Pearson’s correlation analysis, and two-way repeated measures analysis of variance (ANOVA) on the conditions (place, Room Control, Room A, Room B, Room C; time, rest 1, GT, rest 2) were performed. Multiple comparisons using the Tukey–Kramer method were also performed. R (The R Foundation, Vienna, Austria), MATLAB R2018b (MathWorks, Natick, MA, USA), and Microsoft Excel (Microsoft, Redmond, WA, USA) were used for the analysis. The correlation coefficients were roughly defined as |r| = 0.9–1, extremely strong correlation; |r| = 0.7–0.9, strong correlation; |r| = 0.5–0.7, correlation; |r| = 0.3–0.5, weak correlation; and |r| = 0–0.3, virtually no correlation.

## 3. Results

### 3.1. Fatigue-Inducing Experiment

#### 3.1.1. Evaluation of the Pre-Task Fatigue State

Considering the pre-task state as the normal daily fatigue state of the participants, the correlation between the TRS data and the answers of the four subjective tests was analyzed for the 38 participants in rest 1. However, no significant correlation (|r| ≥ 0.5) was found (data not shown). It was hypothesized that this result was attributed to the low average value of the state before the task. The average value of the feeling of fatigue in rest 1 was 43.2. The comparison of states before and after the fatigue-inducing task was considered a more effective means of identifying a fatigue indicator.

#### 3.1.2. Evaluation of the Post-Fatigue-Inducing Task Fatigue State

**Figure 3:**
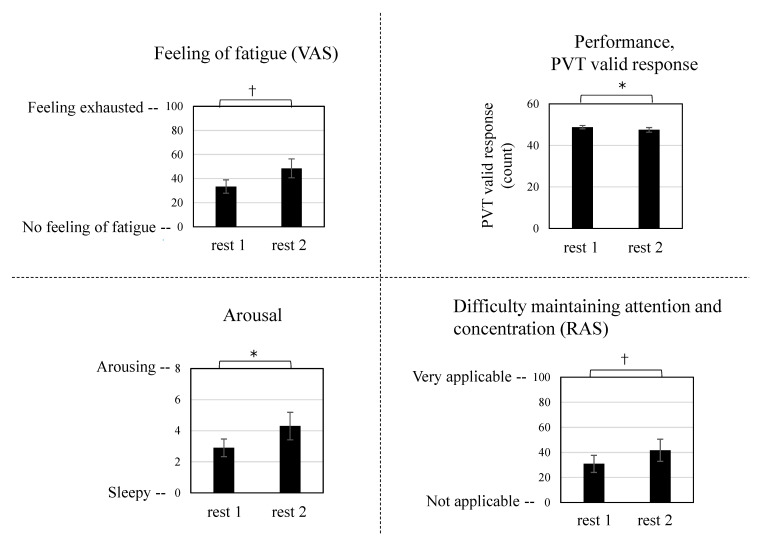
Change in subjective feeling and performance before and after the fatigue-inducing task. Data are expressed as mean ± standard errors (*n* = 10). * *p* < 0.05, † *p* < 0.1. PVT, psychomotor vigilance task; VAS, visual analog scale; RAS, Roken Arousal Scale.

The fatigue states of the participants before and after the fatigue-inducing task were compared. A paired *t*-test was conducted using the results of the subjective tests and the task performance, which were performed before and after the fatigue-inducing task, respectively (Figure 3). Since the TRS data for rest 2 was only for 10 male participants, all subsequent analyses were performed using rest 1 and rest 2 data for these 10 male participants. Significant results (*p* < 0.05) were obtained for the arousing/sleepy value in the Affect Grid test (*p* = 0.034) and the number of valid PVT responses (*p* = 0.033). In addition, the VAS values for the feeling of fatigue (*p* = 0.053) and difficulty maintaining attention and concentration (RAS) test (*p* = 0.057) tended to be significant.

Compared with the results before the fatigue-inducing task, the subjective test results following the task indicated an increase in the VAS value of the feeling of fatigue, arousal, and difficulty maintaining attention and concentration (RAS). The number of valid PVT responses (an objective behavioral indicator) also decreased after the task. From these results, it was inferred that the task effectively induced fatigue in the participants.

#### 3.1.3. Analysis of the Correlation between the VAS Value of the Feeling of Fatigue and TRS Data

**Figure 4:**
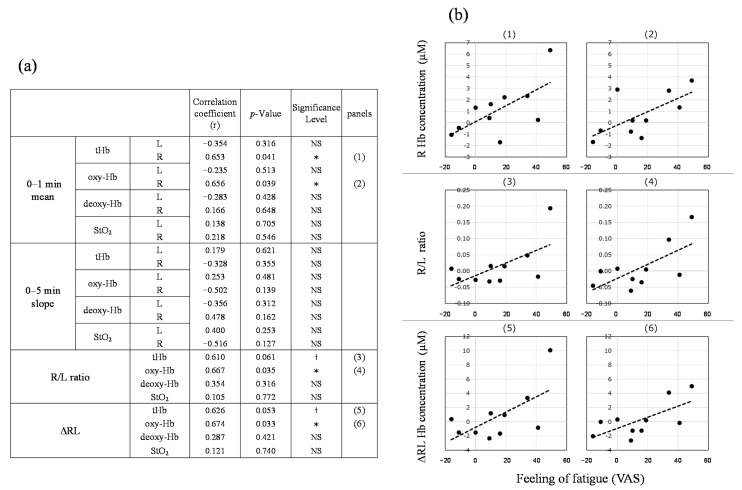
Correlation analysis of the visual analog scale (VAS) of the feeling of fatigue and near-infrared time-resolved spectroscopy (TRS) data. (**a**) Correlation coefficient and (**b**) scatterplot. † *p* < 0.1; * *p* < 0.05. NS, not significant; Hb, hemoglobin; oxy-Hb, oxygenated hemoglobin; deoxy-Hb, deoxygenated hemoglobin; tHb, total hemoglobin; StO_2_, tissue oxygen saturation; L, left prefrontal cortex; R, right prefrontal cortex; R/L ratio, right-left ratio; ΔRL, right-left difference.

The amount of change before and after the task (rest 2 − rest 1) was calculated, and the correlation between the results of the subjective tests, task results, and TRS data was analyzed. No significant correlation was found between the PVT behavioral indicator and TRS data (data not shown). However, a significant correlation (*p* < 0.05) was observed between the feeling of fatigue and TRS data (Figure 4)—specifically, a correlation between the feeling of fatigue and the average tHb and oxy-Hb values in the right prefrontal cortex over 0 to 1 min, the R/L ratio of oxy-Hb and ΔRL oxy-Hb. The highest correlation was found between fatigue feeling and ΔRL oxy-Hb (correlation coefficient, 0.674, *p* = 0.033). Both the R/L ratio of tHb and the difference in tHb between the left and right prefrontal cortices also correlated with fatigue (*p* < 0.1). Because tHb is the sum of oxy-Hb and deoxy-Hb, it was inferred that tHb changed according to the change in oxy-Hb. These results indicated that an increase in fatigue was related to an increase in oxy-Hb in the right prefrontal cortex and in the right-left difference value and R/L ratio.

### 3.2. Indoor Forest Bathing Experiment

#### 3.2.1. Evaluation of Feeling of Fatigue

**Figure 5:**
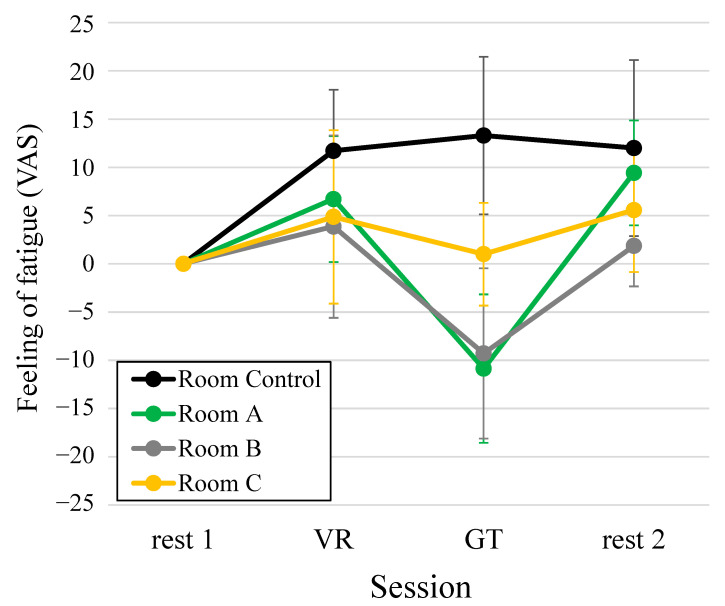
Change in the visual analog scale value of the feeling of fatigue in the prefrontal cortex during the experiment (rest 1, VR, GT, rest 2). Data are expressed as mean ± standard errors (*n* = 7). VR, visual reality; GT, test space; Room Control, space without natural green features; Room A/B/C, space with natural green features.

Analysis was performed using data from seven of the eight participants, excluding one who did not have TRS data for Room C. Figure 5 shows the average VAS value of the feeling of fatigue of the seven participants as a time series variation throughout the experiment. The vertical axis shows the change in the value from rest 1 (each value − rest 1 value), the horizontal axis shows the experimental session, and the legend is the name of each space. In the VR session compared with rest 1 session, the values of feeling of fatigue increased in all the spaces. In Room Control, the values of the feeling of fatigue of the VR, GT, and rest 2 sessions were almost unchanged. In contrast, the value of the feeling of fatigue decreased compared with the VR value when staying in Room A, Room B and Room C. In the remaining two sessions, the value of the feeling of fatigue increased again. However, the ANOVA result showed no significant difference in Figure 5.

#### 3.2.2. Analysis of the Results for Cerebral Blood Flow

**Figure 6:**
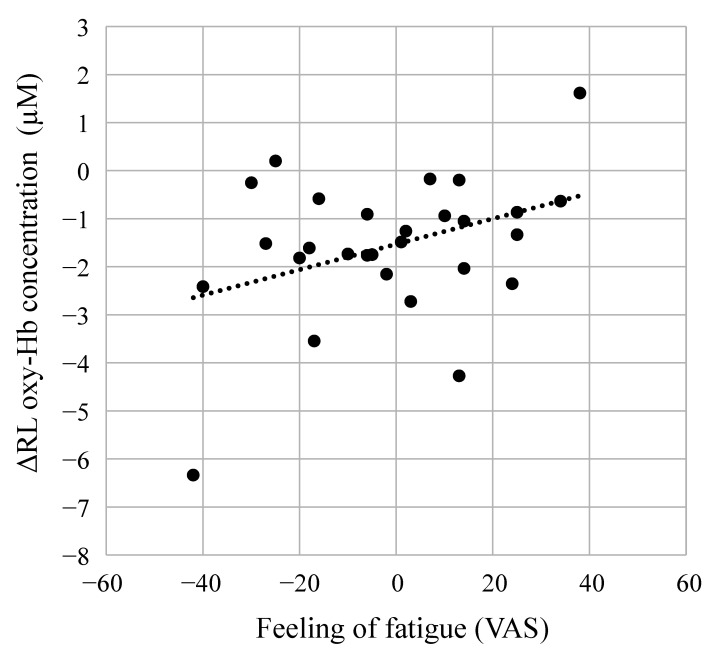
Correlation analysis of feeling of fatigue and time-resolved near-infrared time-resolved spectroscopy data after staying in the experimental spaces. ΔRL oxy-Hb, right-left difference in oxygenated hemoglobin.

We analyzed the indoor forest bathing experiment based on the results of the fatigue-inducing experiment. Figure 4 shows a significant correlation between the value of the feeling of fatigue after the three-back test and ΔRL oxy-Hb in the prefrontal cortex (correlation coefficient, 0.674; *p* = 0.033). Moreover, in this experiment, the changes before and after staying in the room (GT − rest 1) were calculated, and the correlation between the test of the feeling of fatigue and ΔRL oxy-Hb data was analyzed (Figure 6). Since VR is not a high-load task similar to the three-back test, the level of the feeling of fatigue was low at 40; however, a significant correlation was observed between the two (correlation coefficient, 0.374; *p* = 0.040). ΔRL oxy-Hb increased as fatigue increased. We therefore evaluated the effect of staying in indoor green spaces using cerebral blood flow of the seven participants.

**Figure 7:**
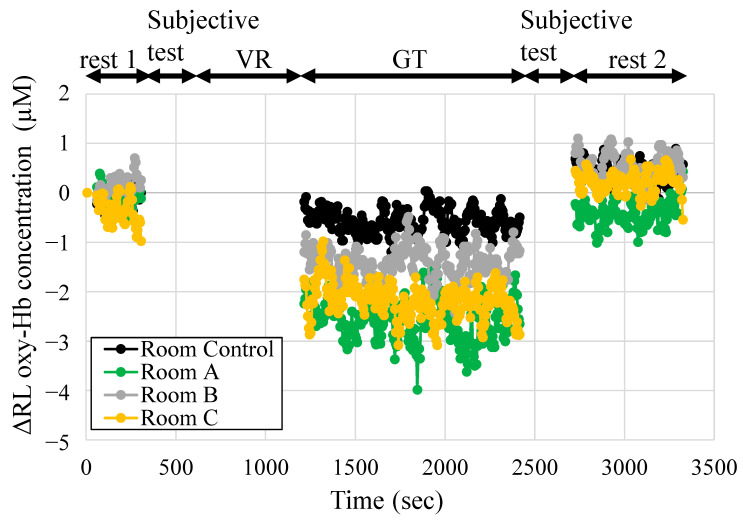
Change in the right-left difference in oxygenated hemoglobin (ΔRL oxy-Hb) in the prefrontal cortex during the experiment (rest 1, GT, rest 2), every 5 s. The vertical axis shows the difference value from 0 to 1-min means of rest 1. Data are expressed as means (*n* = 7). VR, visual reality; GT, test space; Room Control, space without natural green features; Room A/B/C, space with natural green features.

**Figure 8:**
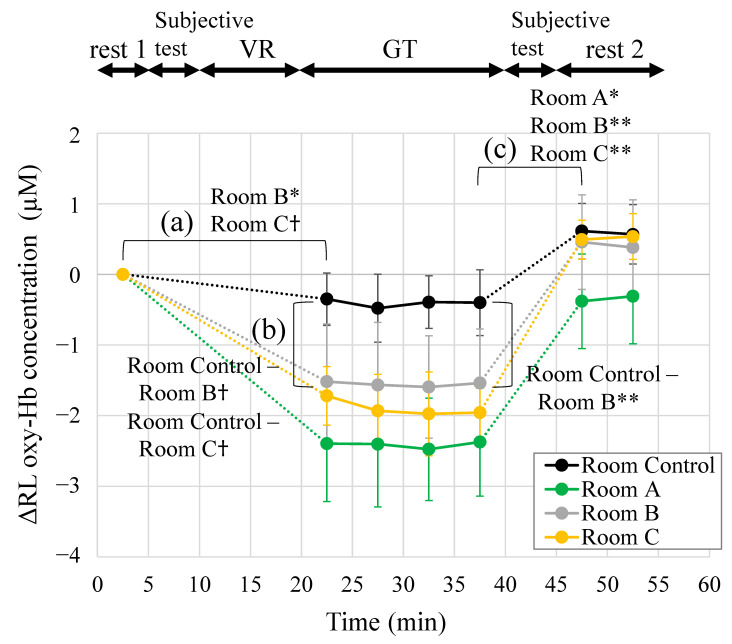
Change in the left-right difference value of the oxygenated hemoglobin (oxy-Hb) in the prefrontal cortex during the experiment (rest 1, GT, rest 2), every 5 min. The vertical axis shows the difference value from rest 1. (**a**) Comparison of rest 1 and the initial 5 min in GT; (**b**) comparison of Room Control and three indoor green spaces, and (**c**) comparison of the last 5 min of GT and rest 2. Data are expressed as mean ± standard errors (*n* = 7). ** *p* < 0.01, * *p* < 0.05, † *p* < 0.1. VR, visual reality; GT, test space; Room Control, space without natural green features; Room A/B/C, space with natural green features.

Figure 7 shows the change in ΔRL oxy-Hb in the prefrontal cortex during rest 1, GT, and rest 2 sessions. Data were measured at 5-s intervals, and the mean value of the seven participants in each space was presented. After entering an indoor green space, ΔRL oxy-Hb decreased compared with rest 1. Room A, Room B, and Room C showed lower values than Room Control. In the rest 2 session, ΔRL oxy-Hb increased again. Each of the 5-min mean values of the ΔRL oxy-Hb in Figure 7 is shown in Figure 8. The vertical axis shows the difference value from rest 1 (each value–rest 1 value), the horizontal axis shows time in the experiment, and the legend is the name of each space. The experimental session is shown at the top of the figure. Multiple comparisons with the Tukey–Kramer method as a result of ANOVA are shown in Figure 8a–c. In Figure 8a, the value of ΔRL oxy-Hb was significantly reduced when staying in Room B (*p* = 0.025). Staying in Room C showed a significant trend (*p* = 0.051). Staying in Room A space showed the lowest value in GT, but the difference was not statistically significant. This was due to the large standard errors between participants, and it was hypothesized that increasing the N number would make a significant difference. In contrast, there was no significant reduction when staying in Room Control. In Figure 8b, Room B and Room C show a decreasing trend (Room Control–Room B, *p* = 0.070; Room Control–Room C, *p* = 0.077) in the initial 5 min of GT session; in the 15–20-min means of GT session with a longer stay, Room B showed significant reduction (Room Control– Room B, *p* = 0.001). ΔRL oxy-Hb significantly increased in Room A (*p* = 0.017), Room B (*p* = 0.007), and Room C (*p* = 0.002).

## 4. Discussion

In this study, we proposed the right-left difference score (ΔRL oxy-Hb), calculated from the absolute value of the oxy-Hb concentration in the prefrontal cortex. It has been reported as right laterality ratio score (RLS = (Right − Left)/(Right + Left) of oxy-Hb and total hemoglobin) for the prefrontal cortex activity under stress [10,11]. Both our proposed score ΔRL oxy-Hb and RLS are indicators related to negative emotions; however, in our experiment, there was no significant correlation between RLS and subjective values of feeling of fatigue. On the other hand, our proposed score ΔRL oxy-Hb showed significant correlation to the subjective values of feeling of fatigue. Since the RLS is an indicator mainly related to stress, it is considered that no significant correlation with feeling of fatigue was observed in our experiment. It is considered appropriate to use our proposed score ΔRL oxy-Hb that calculated from the absolute value of the oxy-Hb concentration in the prefrontal cortex for feeling of fatigue evaluation.

In the fatigue-inducing experiment, when fatigue increased, the oxy-Hb level of the right prefrontal cortex, ΔRL oxy-Hb, and the R/L ratio of oxy-Hb increased. The correlation analysis between all psychological evaluation data and TRS data is shown in Table S1. Table 1 shows the subjective data that were significantly correlated with the TRS data (|r| ≥ 0.5, *p* < 0.05). In all cases, the indicators shown in Table 1 were related to the oxy-Hb in the right prefrontal cortex. Table S2 shows the correlation analysis between the VAS of the feeling of fatigue and the other subjective values. Unrelaxed RAS and unpleasant PSS, which correlated with ΔRL oxy-Hb, had significant correlations with fatigue. This suggests that ΔRL oxy-Hb is also associated with the emotions related to fatigue (unrelaxed and unpleasant).

In the indoor forest bathing experiment, the fatigue feeling value decreased when staying in the Room A, Room B, and Room C spaces. Furthermore, among the subjective evaluations other than the feeling of fatigue, there was a significant difference in the pleasant Affect Grid (Room A, *p* < 0.01; Room B, *p* < 0.01), unpleasant PSS (Room B, *p* < 0.01), fatigue PSS (Room B, *p* < 0.05; Room A, *p* < 0.1), and difficulty in maintaining attention and concentration RAS (Room A, *p* < 0.01; Room B, *p* < 0.01; Room C, *p* < 0.01). There was no significant difference in the unrelaxed RAS, which correlated with ΔRL oxy-Hb showed in Table 1. The result that the ΔRL oxy-Hb level decreased after entering an indoor green space contradicts the increase in the ΔRL oxy-Hb levels that occurred when the participants experienced fatigue. In addition, the pleasant feeling increased and the feeling of fatigue decreased in Room A and Room C. Therefore, it was inferred that spending time in the indoor green spaces had effects of reducing fatigue and improving unpleasant feeling, reflecting a decrease in the ΔRL oxy-Hb levels. Furthermore, distinguishing and evaluating mental fatigue from unpleasantness should be considered in future studies.

**Figure 9:**
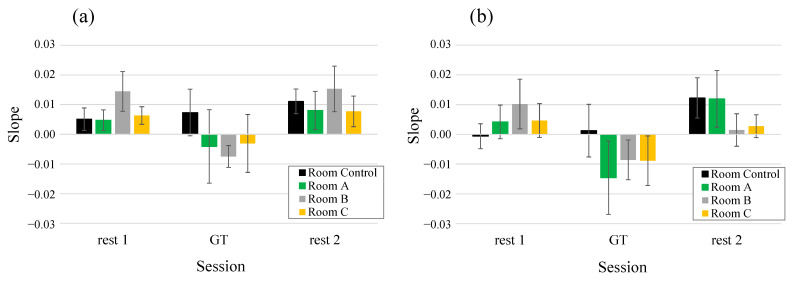
The mean values of the slope for oxygenated hemoglobin in 1-min periods immediately after the start of rest 1, GT, and rest 2 sessions. (**a**) Left prefrontal cortex and (**b**) right prefrontal cortex. Data are expressed as mean ± standard errors (*n* = 7). GT, test space; Room Control, space without natural green features; Room A/B/C, space with natural green features.

In addition, we also considered whether the right or left prefrontal cortex contributed to the change in ΔRL oxy-Hb. In the fatigue-inducing experiment, when fatigue was increased, the oxy-Hb level of the right prefrontal cortex increased significantly, and no significant changes were observed in the left prefrontal cortex. In the indoor forest bathing experiment, Figure 9 shows the average slope values for oxy-Hb in the left and right prefrontal cortices in the 1-min periods immediately following the start of rest 1, GT, and rest 2. The raw data for which the slope was calculated are shown in Figure S1. In the right prefrontal cortex, the slope of the oxy-Hb levels decreased when staying at Room A, Room B, and Room C in the GT session. In contrast, the slope of the oxy-Hb levels in the left prefrontal cortex showed no noticeable change in the GT session. In the indoor forest bathing experiment, a VR session was originally conducted between rest 1 and GT sessions, and the TRS data could not be measured because the headgear was attached. The fatigue feeling value after the VR session increased, but it is not clear whether the oxy-Hb value in the right prefrontal cortex increased following the VR session. However, it was hypothesized that, when tired, the oxy-Hb levels of the right prefrontal cortex increased; thereby, the ΔRL oxy-Hb became large and remained in the indoor green space, causing a decrease in the ΔRL oxy-Hb owing to the decrease in the right prefrontal cortex. In particular, the differences in the plant types (Room A, Room B, and Room C) had no effect on TRS data, and it was hypothesized that they had similar fatigue recovery and relaxation effects. It is hoped that differences in the effects of the three rooms will be explained more clearly in the future.

Some experiments reported a decrease in the oxy-Hb concentration in the prefrontal cortex, owing to observations of natural phenomena, and the effects on the left and right sides of the prefrontal cortex differed. Viewing a forest image and fresh roses caused a decrease in the right side of the prefrontal cortex [5,6], and touching Hinoki and working with foliage plants caused a decrease in the left side of the prefrontal cortex [7,8]. In our experiment, the right side of the prefrontal cortex decreased in oxy-Hb concentration, similar to the result of the forest image and fresh roses experiments, in which visual and olfactory modalities are presumed to be involved. We hope that future research will reveal which stimuli are associated with reduced cerebral blood flow in the prefrontal cortex.

Our experiments identified a correlation between the fatigue feeling before and after a task and the amount of change in the cerebral activity of oxy-Hb in the right prefrontal cortex. This indicates that the changes in the cerebral blood flow in the right prefrontal cortex reflect fatigue. A previous study on brain functions also discussed the possibility that right-left differences in brain wave activity in the frontal region (alpha waves in the frequency range from 8 to 12 Hz) may reflect comfort/discomfort. It has also been reported that activity is prioritized on the right side of the brain when discomfort is felt and that activity is prioritized on the left side of the brain when comfort is felt [20,21]. In addition, other reports have suggested a connection between the right side of the brain and avoidance (i.e., the tendency to move away and escape from a stimulus) and the left side of the brain and proximity (i.e., the tendency to move closer and acquire a stimulus) [22]. These findings suggest that an association exists between fatigue and the increase in the changes in the oxy-Hb cerebral activity in the right prefrontal cortex, as identified in this experiment. Moreover, our results that the increase in the oxy-Hb levels in the right prefrontal cortex reflected unpleasant feelings might correspond to those of previous studies.

On the other hand, the specific regions within the prefrontal cortex that respond to the reduction of oxy-Hb in the right frontal lobe due to the green effect have been unclear. Recently, it has been reported that, using functional near-infrared spectroscopy, the oxy-Hb concentration in the right orbitofrontal cortex significantly decreased while viewing images of nature [23]. The orbitofrontal cortex integrates information from the sensory cortex, autonomic areas of the brainstem, and the amygdala and plays an essential role in value expression and emotional processing [24]. It is also involved in the pathophysiology of depression and anxiety [25,26]. Our results, showing significant decrease in the oxy-Hb concentration in the right frontal cortex may reflect changes in the orbitofrontal cortex. This also corresponds to a previous study mentioned above that showed a significant decrease in orbitofrontal cortex while viewing images of the nature, which may reflect the effect of indoor forest reproduction.

In this study, our proposed score for ΔRL oxy-Hb was shown to increase in association with feeling of fatigue and unpleasant emotions, and decrease in association with improvement of feeling of fatigue and unpleasant emotions. However, since adequately verifying any hypothesis on brain wave activity is challenging [27], we hope that this phenomenon is more clearly explained in the future.

## 5. Conclusions

The fatigue-inducing task experiment identified a significant correlation between the amount of change in the cerebral blood flow and fatigue. The results of the experiment suggest that the increase in the oxy-Hb levels in the right prefrontal cortex, ΔRL oxy-Hb, and R/L ratio in the prefrontal cortex are related to the feeling of fatigue. Furthermore, the ΔRL oxy-Hb was found to have an inverse correlation with feelings of relaxation and comfort. Using ΔRL oxy-Hb as a candidate for a fatigue indicator, this experiment verified the fatigue recovery effect of spending time in indoor forest bathing environment created under laboratory conditions. The following conclusions were drawn:

1. Subjective evaluations: The feeling of fatigue was lower after spending time in the Room A, Room B, and Room C green spaces than after spending time in Room Control without green features. In addition, pleasant feelings increased and fatigue PSS decreased in Room A and Room C.
2. Changes in cerebral blood flow: ΔRL oxy-Hb was lower after spending time in Room A, Room B, and Room C green spaces than in Room Control. This was an opposite result to the increase in ΔRL oxy-Hb that related to the feeling of fatigue. Therefore, it was hypothesized that the decrease in the ΔRL oxy-Hb corresponded to the reduction in fatigue and improvement of pleasant feeling.

Physiological and psychological changes were observed when staying in the indoor green space constructed in the biotron greenhouse, where plant groups were reproduced similar to those in nature.

## Figures and Tables

**Table 1:** Significant correlation between the subjective test and time-resolved spectroscopy data in the fatigue-inducing experiment.

Correlation Coefficient (r)	R 0–1-min Means	R/L Ratio	ΔRL	R 0–5-min Slope
VAS of feeling of fatigue	0.656	0.667	0.674	NS
Unrelaxed RAS	NS	NS	0.658	NS
Unpleasant PSS	NS	NS	0.658	NS
Anxiety PSS	NS	NS	NS	−0.714
Strain PSS	0.681	NS	NS	NS

R, right; R/L Ratio, right-left ratio; ΔRL, right-left difference; VAS, visual analog scale; RAS, Roken Arousal Scale; PSS, Phasic Stress Scale; NS, not significant.

## Data Availability

The data presented in this study are available on request from the corresponding author upon reasonable request.

## Supplementary Materials

The following supporting information can be downloaded at: https://www.mdpi.com/article/10.3390/ijerph19116672/s1, Figure S1: The slope in the oxy-Hb concentration in the prefrontal cortex during the experiment, Table S1: Significant correlation between subjective test and TRS data, Table S2: Correlation analysis of the visual analog scale value of the feeling of fatigue and another subjective test after the fatigue-inducing task.
