# Supplementary material for: Effect of Indoor Forest Bathing on Reducing Feelings of Fatigue Using Cerebral Activity as an Indicator
Source: Int J Environ Res Public Health. 2022 May 30;19(11):6672. doi: 10.3390/ijerph19116672 (PMC9180409; doi:10.3390/ijerph19116672)
Supplement: Supplementary file 1 [file ijerph-19-06672-s001.zip › ijerph-1672606-supplementary.pdf]

International Journal of
Environmental Research and Public Health

MDPI

Supplementary Materials

Table S1. Significant correlation between subjective test and near-infrared time-resolved spectroscopy data.

			Affect Grid		RAS						PSS					
			Pleasant	Arousing	Sleepiness	Unactive	Unrelaxed	Strain (tension)	Difficulty maintaining attention and concentration	Lack of motivation	Malaise	Unpleasant	Angry	Anxiety	Stress	Strain
0-1 min mean	tHb	L	0.306	0.255	−0.142	0.359	−0.448	−0.072	0.279	−0.304	−0.210	−0.513	0.218	0.191	−0.334	−0.243
		R	−0.227	−0.319	0.105	−0.572	0.334	0.073	−0.300	0.571	0.451	0.412	0.021	0.354	0.382	0.602
	oxy-Hb	L	0.221	0.059	−0.184	0.059	−0.444	0.193	−0.001	−0.177	−0.097	−0.411	0.239	−0.060	−0.034	0.074
		R	−0.580	−0.094	0.000	−0.461	0.494	0.363	−0.427	0.323	0.610	0.531	0.208	0.184	0.328	**0.681**
	deoxy-Hb	L	0.231	0.335	−0.021	0.472	−0.195	−0.326	0.419	−0.278	−0.224	−0.333	0.065	0.369	−0.479	−0.463
		R	0.432	−0.410	0.182	−0.300	−0.134	−0.391	0.103	0.501	−0.105	−0.057	−0.265	0.320	0.172	0.043
	StO_2	L	−0.105	−0.244	−0.104	−0.389	−0.060	0.390	−0.364	0.155	0.173	0.042	0.111	−0.328	0.402	0.461
		R	−0.619	0.236	−0.152	−0.135	0.325	0.483	−0.373	−0.139	0.399	0.280	0.267	−0.180	0.076	0.377
0-5 min slope	tHb	L	−0.424	−0.363	0.176	0.033	0.369	0.312	−0.190	0.014	0.194	0.463	−0.097	−0.597	0.218	0.208
		R	0.007	−0.490	0.522	0.412	−0.015	−0.349	0.389	0.279	−0.202	−0.041	−0.616	**−0.867**	−0.064	−0.269
	oxy-Hb	L	−0.300	−0.416	0.193	−0.097	0.299	0.323	−0.183	0.115	0.179	0.430	−0.096	−0.479	0.343	0.308
		R	0.220	−0.162	0.130	0.231	−0.269	−0.227	0.203	0.078	−0.309	−0.260	−0.352	**−0.714**	0.035	−0.310
	deoxy-Hb	L	−0.281	0.357	−0.119	0.479	0.100	−0.174	0.074	−0.359	−0.021	−0.071	0.027	−0.200	−0.536	−0.448
		R	−0.371	−0.297	0.389	0.078	0.448	−0.023	0.102	0.195	0.291	0.398	−0.124	0.209	−0.138	0.214
	StO_2	L	−0.143	−0.461	0.198	−0.289	0.252	0.288	−0.141	0.288	0.193	0.382	−0.061	−0.234	0.532	0.436
		R	0.287	0.072	−0.086	0.200	−0.354	−0.103	0.133	−0.081	−0.297	−0.305	−0.104	−0.496	0.075	−0.290
R / L ratio (0-1 min mean)		tHb	−0.281	−0.383	0.153	−0.571	0.436	0.065	−0.305	0.581	0.402	0.498	−0.104	0.101	0.479	0.531
		oxy-Hb	−0.509	−0.213	0.140	−0.472	0.595	0.146	−0.324	0.460	0.517	0.611	−0.021	0.151	0.361	0.541
		deoxy-Hb	0.075	−0.464	0.107	−0.527	0.127	−0.073	−0.192	0.555	0.142	0.204	−0.172	0.012	0.478	0.341
		StO_2	−0.492	0.372	−0.080	0.157	0.356	0.143	−0.070	−0.234	0.245	0.213	0.179	0.068	−0.208	−0.002
ΔRL (0-1 min mean)		tHb	−0.318	−0.386	0.150	−0.548	0.467	0.091	−0.330	0.538	0.416	0.540	−0.089	0.106	0.441	0.527
		oxy-Hb	−0.588	−0.162	0.116	−0.391	**0.658**	0.176	−0.324	0.373	0.550	**0.658**	0.037	0.164	0.299	0.507
		deoxy-Hb	0.135	−0.483	0.133	−0.476	0.038	−0.054	−0.183	0.497	0.075	0.166	−0.205	−0.019	0.406	0.313
		StO_2	−0.504	0.363	−0.058	0.161	0.358	0.164	−0.073	−0.228	0.257	0.235	0.174	0.067	−0.209	0.024

Bold and gray hatch: $|r| > 0.5$ and $p < 0.05$.

RAS, Roken Arousal Scale; PSS, Phasic Stress Scale; Hb, hemoglobin; oxy-Hb, oxygenated hemoglobin; deoxy-Hb, deoxygenated hemoglobin; tHb, total hemoglobin; StO_2, tissue oxygen saturation; R, right; L, left; R/L ratio, right-left ratio; ΔRL, right-left difference.

Table S2. Correlation analysis of the visual analog scale value of the feeling of fatigue and another subjective test after the fatigue-inducing task.

		Correlation coefficient (r)	*p*-value	Significance level
Affect Grid	Pleasant	–0.566	0.088	†
	Arousing	–0.261	0.466	NS
RAS	Sleepiness	0.049	0.892	NS
	Unactive	–0.306	0.390	NS
	Unrelaxed	0.724	0.018	*
	Strain (tension)	0.461	0.180	NS
	Difficulty maintaining attention and concentration	–0.116	0.749	NS
	Lack of motivation	0.395	0.259	NS
PSS	Malaise	0.788	0.007	**
	Unpleasant	0.719	0.019	*
	Angry	0.533	0.113	NS
	Anxiety	0.419	0.229	NS
	Stress	0.757	0.011	*
	Strain	0.822	0.004	**

† $p < 0.1$, *$p < 0.05$, **$p < 0.01$.
NS, not significant; RAS, Roken Arousal Scale; PSS, Phasic Stress Scale.

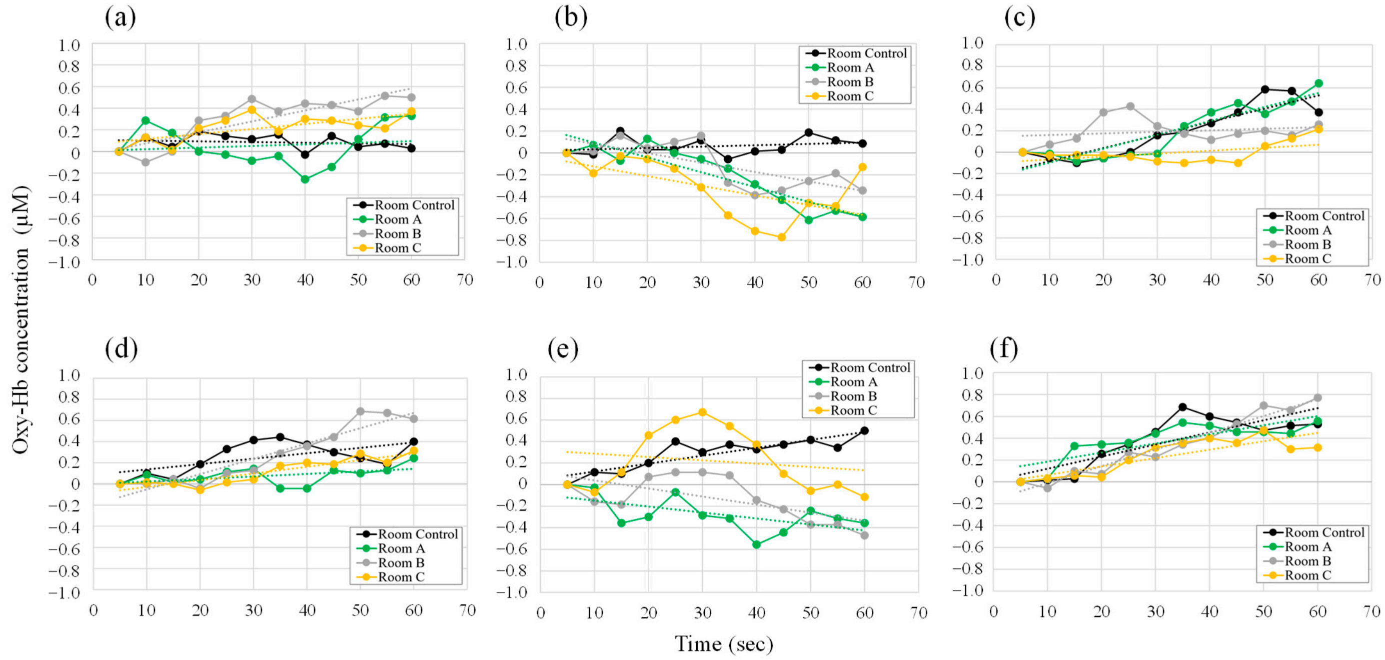

Figure S1. The slope in the oxygenated hemoglobin concentration in the prefrontal cortex during the experiment, every 5 s over a 60-s period. The vertical axis shows the difference value from the first 5 s. **(a–c)** Right prefrontal cortex, **(d–f)** left prefrontal cortex, **(a and d)** rest 1, **(b and e)** GT, and

(c and f) rest 2. Data are expressed as means (*n* = 7). Oxy-Hb, oxygenated hemoglobin; Room Control, space without natural green features; Room A/B/C, space with natural green features.
